# Women’s experiences giving birth outside of health facilities in Kenya during the COVID-19 pandemic: a qualitative study

**DOI:** 10.1136/bmjopen-2025-101458

**Published:** 2025-09-15

**Authors:** Rebecca Woofter, Krupa Varghese, John Mboya, Ginger Golub, May Sudhinaraset

**Affiliations:** 1 Community Health Sciences, University of California Los Angeles, Los Angeles, California, USA; 2 Innovations for Poverty Action Kenya, Nairobi, Kenya

**Keywords:** OBSTETRICS, COVID-19, Person-Centered Care, Pregnant Women, Postpartum Women

## Abstract

**Abstract:**

**Objectives:**

The COVID-19 pandemic disrupted maternal healthcare access globally, leading many women to give birth outside of healthcare facilities, often assisted by traditional birth attendants (TBAs). This study explored the experiences of Kenyan women who gave birth outside of healthcare facilities during the COVID-19 pandemic based on the Person-Centered Maternity Care (PCMC) framework.

**Design/setting:**

This study used data from descriptive qualitative indepth interviews with Kenyan women who gave birth outside of healthcare facilities between March and November 2020. Participants lived within the catchment areas of six health facilities in Kiambu and Nairobi counties and were recruited by community leaders and community health workers. Interviews were conducted in 2020 via phone and inductively coded and analysed by a team of researchers.

**Participants:**

A total of 28 Kenyan women who gave birth outside of healthcare facilities between March and November 2020 completed indepth interviews.

**Results:**

In this sample, approximately one-third of women were assisted by TBAs, while the majority were supported by friends and family members. Women generally described salient aspects of their care pertaining to the PCMC domain of supportive care. During labour, birth and the immediate postpartum, women received emotional support, basic medical assistance and instrumental support such as preparing food and baths. However, women also described concerns about giving birth outside of healthcare facilities, including poor hygiene and lack of privacy in birthing spaces as well as instances of verbal and physical harassment by TBAs. Overall, women worried about potential complications during birth, and many women delayed seeking postpartum and postnatal care.

**Conclusion:**

Women who were unable to access healthcare facilities during the COVID-19 pandemic relied on TBAs and/or friends and family for support during labour and birth. These women faced gaps in PCMC, specifically related to supportive care. Given that birthing outside of healthcare facilities remains common globally, particularly during emergencies such as pandemics, TBAs should be supported to provide more person-centred care to women giving birth outside of healthcare facilities.

**STRENGTHS AND LIMITATIONS OF THIS STUDY**

- This is the first study to our knowledge to use the Person-Centered Maternity Care framework to examine women’s experiences giving birth outside of health facilities.
- This study used qualitative indepth interviews to explore the experiences of women who gave birth with friends or family members as well as those who gave birth with traditional birth attendants during the COVID-19 pandemic in Kenya.
- Most women in this sample intended to give birth at healthcare facilities and unexpectedly gave birth elsewhere, and their experiences may differ from those who intended to give birth outside of healthcare facilities.

## Introduction

 The COVID-19 pandemic impacted healthcare access globally, resulting in decreased use of preventive healthcare, such as cancer screenings and childhood vaccinations, as well as interruptions to maternity care services.^1^,^5^ Decreased utilisation of healthcare during pregnancy, labour and birth and postpartum puts women and infants at risk of preventable morbidity and mortality.^6 7^ In sub-Saharan Africa, with some of the highest rates of maternal and infant mortality worldwide, barriers to maternity care are of particular concern.^8 9^ Across sub-Saharan Africa, women had lower rates of attending antenatal care, delivering at health facilities and receiving postpartum and postnatal care during the pandemic than prior to the pandemic.^10^,^14^

In Kenya, initial measures were imposed to address the COVID-19 pandemic on 16 March 2020. These measures restricted travel, closed schools and workplaces and instituted a nightly curfew.^15 16^ During the COVID-19 pandemic, healthcare capacity was strained, including limitations on available staff and supplies in healthcare facilities.^17^ As in other sub-Saharan African countries, women in Kenya attended fewer antenatal care visits and more commonly delivered outside of healthcare facilities during the pandemic than before the pandemic due to multilevel barriers to healthcare.^1314 18^,^22^ Many Kenyan women who gave birth outside of healthcare facilities turned instead to traditional midwives or traditional birth attendants (TBAs) for support.^2023^,^25^ While traditional midwives generally attend an educational midwifery programme, TBAs generally gain their knowledge through apprenticeship and experience assisting women.^26 27^ Both traditional midwives and TBAs support women during labour and birth by providing comfort and encouragement, catching the baby, delivering and disposing of the placenta and preparing baths and meals.^27 28^

Notably, even prior to the COVID-19 pandemic, births outside of healthcare facilities were common. In low- and middle-income countries, about 28% of births occur outside of healthcare facilities, with the highest rates in sub-Saharan Africa.^29^ Non-facility births have been decreasing over time,^30^ though nearly 40% of women in Kenya gave birth outside of healthcare facilities in 2012,^29^ down from 53% in 2008.^31^ Indeed, some Kenyan women prefer to give birth with TBAs rather than at healthcare facilities due to negative experiences with the healthcare system as well as the increased accessibility of TBAs and ability to make payments over time with TBAs.^32 33^ TBAs are also known to provide emotional support and instrumental support, including chores and cooking, which women may not receive in healthcare facilities.^28 32^ Still, both women and TBAs acknowledge concerns that TBAs lack the supplies and expertise to manage potential medical complications during labour and birth.^28 32 34 35^ This study does not specifically encourage births outside of health facilities in Kenya, but rather seeks to examine and document the experiences of a sample of women who gave birth outside of health facilities in a unique context.

Whether in healthcare facilities or with traditional midwives or TBAs, healthcare provided during labour and birth should be person-centred for all women. Person-centred care respects and responds to the needs and preferences of the recipient.^36^ Person-Centered Maternity Care (PCMC) (including care during both labour and birth) is associated with reduced maternal and newborn complications and increased willingness to seek care in the future.^37 38^ A scale for PCMC was developed and validated in Kenya, capturing three domains: dignity and respect, communication and autonomy and supportive care.^39^ The PCMC framework is a useful way to assess the quality of healthcare provided during labour and birth. While the PCMC scale has thus far only been used to examine the quality of care provided within healthcare facilities, the domains may also apply to care experiences outside of healthcare facilities. Despite the frequency of births outside of healthcare facilities in sub-Saharan Africa, few studies have examined women’s experiences during these births, including the experiences of women assisted by traditional midwives or TBAs.^40^,^42^ It is important to assess the extent to which care provided by traditional midwives or TBAs is person-centred and subsequently to develop interventions to improve the quality of care women receive in these settings.

Many women gave birth outside of healthcare facilities in Kenya during the COVID-19 pandemic due to issues with transportation, being turned away from healthcare facilities and fear of contracting COVID-19.^23 25^ This study builds on prior work by examining the experiences of those women who initially planned to deliver at a healthcare facility, yet gave birth outside of health facilities due to one or more of these barriers. Given the potential for future emergency situations that divert pregnant women from healthcare facilities, it is important to understand the experiences of these women and identify ways to improve healthcare access and quality for labour and birth in the future. This study aims to expand the existing literature by examining the experiences of women in Kenya who gave birth outside of healthcare facilities during the COVID-19 pandemic through the lens of PCMC.

## Methods

### Recruitment

The current study was a descriptive qualitative supplement^43^ to a quantitative parent survey exploring the experiences of women in Nairobi and Kiambu Counties who gave birth in 2020 after the imposition of COVID-19 restrictions by the Kenyan government (16 March 2020). Details on the survey have been published previously.^1944^,^46^ Eligible participants for the survey included women aged 15–49 years who had singleton births, resided within the catchment areas surrounding six participating health facilities in Nairobi and Kiambu Counties and possessed a functional mobile phone to allow for remote data collection. Among survey respondents, those who gave birth outside of healthcare facilities were eligible for the qualitative supplement. Participant recruitment took place through engaging community leaders and community health volunteers. Women identified as potentially eligible for the study were asked if they wanted to hear more about the study and if their contact information could be shared with the researchers. All study procedures conformed to the ethical principles embodied in the Declaration of Helsinki and were reviewed and approved by the Institutional Review Board at the University of California, Los Angeles (IRB #20–0 01 421) as well as the Kenya Medical Research Institute. The description of the methods and results of this study aligns with the Consolidated Criteria for Reporting Qualitative Research checklist, as provided in online supplemental table S1. The collaboration of the research team members between the USA and Kenya is described in online supplemental table S2.

### Indepth interviews

Data collection for the parent survey occurred between September and November 2020. Of the 1100 women who participated in the survey, 46 reported giving birth outside of health facilities and were thus the focus for the qualitative supplement. These 46 women were contacted by mobile phone up to nine times to determine their interest in completing indepth interviews. Overall, 28 women completed interviews in November and December 2020. All interviews were conducted via telephone in Kiswahili or English, based on the participant’s preference, by one female researcher who was trained on virtual qualitative interview methods. The interviewer was in a private place for all interviews and participants were asked to do the same. At the beginning of each interview, the interviewer told participants that the goal of the study was to learn about their experience giving birth outside of a health facility during the COVID-19 pandemic, confirmed participants’ eligibility and obtained verbal consent to participate in the study and to audio record the interview. Although the interviewer did not know the participants previously, she worked to build rapport throughout the interview. During the interviews, the interviewer followed a semistructured field guide to ask women about their experiences during pregnancy, labour and birth and post partum (see online supplemental materials). The interviewer also took field notes during the interviews. Each interview lasted 45–60 min. All women who consented and completed any part of the interview received Ksh 150 or the equivalent of approximately $1.40 in Airtime. Following the interviews, the recordings were translated into English, if applicable, and transcribed and deidentified for analyses.

### Analysis

All interview transcripts were inductively coded by a team of four coders with training in public health. Three of the coders lived in Kenya and were also involved in data collection. The fourth coder lived in the USA and joined the project at the analysis phase. All coders received training on qualitative research and maternal and child health. Coding proceeded using a four-phase approach. In the first phase, two coders each read the same six transcripts and created a list of codes based on the content of the interviews, and a third coder read the two sets of coded transcripts and reconciled them, creating a first draft of the codebook. In the second phase, three coders each coded five separate transcripts using the drafted codebook, while the fourth coder reviewed all fifteen transcripts and made adjustments to the applied codes as needed. Three transcripts were blind-coded by two coders during this phase to calculate inter-rater reliability. The two coders achieved 80% agreement across these three transcripts. At the end of this phase, the codebook was updated with the newly created codes to capture additional content from the second phase of transcripts. In the third phase, one coder read and independently coded the final seven transcripts. Very few codes were added in the third phase, suggesting that code saturation was reached.^47^ Finally, in the fourth phase, one coder reviewed all 28 transcripts to ensure the final codebook was consistently applied across all transcripts. The entire coding team met regularly throughout the coding process to discuss any questions and disagreements. Coding was conducted in Dedoose. The final codebook was organised by topics (eg, pregnancy, birth, post partum) and included both parent codes (eg, birth plan, experience with home birth, postpartum care use) and child codes (eg, planned to deliver at private facility, lack of pain management during birth, postpartum care delayed due to recovery from birth).

After all transcripts were coded, the authors reviewed relevant excerpts pertaining to women’s experiences during labour and birth as well as healthcare access in the following days and weeks. The authors identified themes using thematic analysis.^48 49^ During analyses, particular consideration was given to codes and themes reflecting the domains of PCMC (dignity and respect, communication and autonomy, supportive care).^36 39^ Definitions for each domain are shown within table 1. While the semistructured field guide was not developed to specifically capture each PCMC domain, the codebook ultimately reflected several aspects of women’s experiences that indicated the extent to which the care they received was person-centred. During analyses, the authors organised the inductive codes within the domains of PCMC as applicable. For example, codes relating to harassment by TBAs reflected the domain of dignity and respect and codes relating to the cleanliness of the birth environment and encouragement during labour both reflected the domain of supportive care, as shown in table 1. Given the different experiences women reported based on whether or not they were supported by a TBA during labour and birth, findings were stratified by type of support persons (TBAs vs friends/family). The researchers identified consistent themes across participants in each group, suggesting saturation in the key themes regarding participants’ experiences during labour and birth.^47 50^

**Table 1:** Descriptions and examples for each Person-Centered Maternity Care (PCMC) domain*

PCMC domain	Description	Example of PCMC	Example of lack of PCMC
Dignity and respect	Healthcare providers treating patients with respect and ensuring patient privacy and confidentiality Lack of dignity and respect includes instances of verbal or physical harassment	–	“*She was harassing me at some point…my energy that I was left with, I feel like it was deteriorating. I was with no energy and she was like ‘you should just push, you should push.’ I was telling her to help but she wanted even to slap me.”*
Communication and autonomy	Healthcare providers fully explaining tests or procedures and answering questions in an understandable way Healthcare providers allowing patients to make fully-informed decisions regarding their care	–	–
Supportive care	Healthcare providers supporting patients during labour and birth, including addressing fears and pain Cleanliness, privacy and safety in the facility and availability of appropriate staff and supplies in the facility Level of trust in healthcare providers and the care they provide	“*I trusted her [TBA] because she usually assists a lot of women. I was not therefore scared at that time.”* “*She gave me strength and told me that I won’t have problems and I believed that I will give birth well…She was helping me by asking how I was feeling, and she was near me comforting me and telling me even if it’s painful just to persevere.”*	*“It is just a temporary room for giving birth and the blanket that you sleep on, you can’t tell when it was washed…There [with the TBA] you share the same room. A woman sits on one side and you on one side, so when you give birth, the midwife [TBA] serves both of you.”*

* Descriptions based on Sudhinaraset *et al*^36^ and Afulani *et al*.^39^

TBA, traditional birth attendant.

### Patient and public Involvement

Patients were recruited to participate in this study, but were not specifically involved in the design, implementation or dissemination of the study.

## Results

This section first provides an overview of the participants’ demographic characteristics. Themes are then described, first for those who received assistance from TBAs, followed by those who received assistance from support persons such as friends, family members and husbands. Some women received assistance from both TBAs and support persons and are thus included in both sections. Among those who gave birth with TBAs, three themes were identified: (1) selection of and trust in TBAs, (2) experience of care with TBAs and the PCMC domains these experiences reflect and (3) complications of birth and delayed access to postpartum and postnatal care. Among those who gave birth with support persons, three themes were identified: (1) experience of care with support persons, (2) husbands’ involvement during labour and birth and (3) complications of birth and delayed access to postpartum and postnatal care.

### Characteristics of study participants

The 28 women in this study ranged in age from 17 years to 45 years and were on average 32 years old. Most women were married and had two to five children. Of those with multiple children, all reported having previously delivered at a healthcare facility, while only three women reported ever previously giving birth outside of healthcare facilities. For their most recent birth, all but one woman intended to give birth at a healthcare facility and unexpectedly gave birth outside of a healthcare facility. The specific circumstances leading to these births outside of healthcare facilities have been previously described.^23^

A total of 11 women in the study were assisted by TBAs at some point during labour, birth and/or immediately following birth. Of this group, one woman was alone during birth and was assisted by the TBA after giving birth. Overall, 21 women reported receiving assistance from support persons such as friends, family members or husbands during labour, birth and/or immediately following birth. Of those who received assistance from support persons, eight were alone during the actual birth and then were supported after birth. Five women were assisted both by TBAs and support persons. Only one woman in the study reported receiving no assistance at any time. This woman had previously given birth alone at home and did not feel she could call anyone to help her at the time. While most women were at their own homes when they gave birth, some went to TBAs’ birthing facilities and a few were at friends’ houses.

### Experience giving birth with TBAs

#### Selection of and trust in TBAs

Women assisted by TBAs during labour and birth reported multiple reasons for choosing that specific TBA. A majority of these women indicated they had an existing relationship with the TBA or knew others who had gave birth with the TBA.

We heard from my neighbours and the people of that village that…she [TBA] helps people deliver well. I have known her for about five years. (Age 40+ years, three children)

Women who chose the TBAs based on previous relationships noted that this helped them trust the TBA, which is an aspect of supportive care. Conversely, women who did not know the TBA when they gave birth did not necessarily trust the TBA.

I trusted her [TBA] because she usually assists a lot of women. I was not therefore scared at that time. (Age 20–29 years, four children)

#### Experience of care with TBAs and PCMC domains

In addition to assisting women throughout labour and birth, TBAs also assisted women immediately following birth by cutting the umbilical cord, delivering the placenta and cleaning the baby. A few women also indicated that the TBAs prepared food and baths for them. A majority of the women assisted by TBAs reported satisfaction with the care they received from the TBA, as the TBAs assisted them to safe and successful births. Women described specific aspects of their experience with TBAs which aligned with the PCMC domains. About half of the women reported that the TBA provided encouragement and emotional support, reflecting supportive care.

She gave me strength and told me that I won’t have problems and I believed that I will give birth well…She was helping me by asking how I was feeling, and she was near me, comforting me, and telling me even if it’s painful just to persevere. (Age 30–39 years, five children)

However, many women also reported aspects of their experience which did not reflect person-centred care. Although many women reported receiving encouragement from TBAs, a few women noted that they were harassed by TBAs, which indicates a lack of respectful care.

She was harassing me at some point…My energy that I was left with, I feel like it was deteriorating. I was with no energy and she was like ‘you should just push, you should push.’ I was telling her to help but she wanted even to slap me. (Age <20 years, one child)

Additionally, women described concerns with the lack of comprehensive services offered by TBAs, which suggests a lack of supportive care.

When you give birth from the hospital, the nurse will wash the child, but the traditional birth attendants don’t wash the babies. Mostly, they [TBAs] just wrap them in your lesso [wrap for carrying babies] and place them beside you. (Age 30–39 years, three children)

Indeed, about half of the women who gave birth with TBAs felt fear and stress during labour and birth as they knew that TBAs had limited ability to manage any complications that may arise.

I felt like the baby may die or even myself. The pregnancy may require an operation and at home, it is not possible…isn’t that just death? At the hospital they may know if you require an operation, but when you are at home all those things are not there. So, I was afraid. I was afraid, but I left it all to God. (Age 30–39 years, four children)

Finally, women also described concerns with the physical environment in birthing spaces with TBAs, which also suggested a lack of supportive care. In particular, some women were dissatisfied with the lack of sufficient cleanliness and hygiene in these spaces, reporting that the sheets provided were rough and dirty and that the birthing spaces were not well-maintained. One woman also mentioned a lack of privacy with the TBA, as there were multiple women being assisted in the birthing space at once.

It is just a temporary room for giving birth and the blanket that you sleep on, you can’t tell when it was washed. But in the hospital, it is always white and it is usually changed… It is not like the hospital where there is privacy with a doctor. There [with the TBA], you share the same room. A woman sits on one side and you on one side so when you give birth the midwife [TBA] serves both of you. (Age 30–39 years, four children)

#### Complications of birth and delayed access to postpartum and postnatal care

The only complications reported by women who gave birth with TBAs were excessive pain and bleeding during and after birth. However, a majority of women who gave birth with TBAs went two or more days before accessing postpartum or postnatal care at healthcare facilities. Of these, half accessed postpartum or postnatal care after 1 week, a few after 2 weeks and one woman did not receive care for more than 1 month.

There was a difference [between delivering at a healthcare facility versus with a TBA] because if you give birth there [hospital], the baby’s weight is taken immediately and [the baby is] given the tetanus vaccine…When I gave birth to this one, however, I stayed with him for two weeks before taking him [to a doctor]. (Age 20–29 years, four children)

Some women reported delays in accessing postpartum and postnatal care due to pain following birth, and others expressed fear of judgement from healthcare providers.

Let me tell you, if you give birth at home, you will have problems with the doctors. You shouldn’t go with a complication and say that you gave birth at home. They won’t understand you. You would not have a chance to explain what made you give birth at home…It is that fear that made me not go [for postpartum care]. (Age 30–39 years, five children)

### Experience giving birth with support persons

#### Experience of care with support persons

Most women in the sample described being supported by friends and family members during and after giving birth. In most cases, these support persons cut the umbilical cord and provided instrumental support, including cleaning, cooking and caring for older children in the household.

At 4am, she [a friend] came and prepared water and I showered. She asked whether there was anything that remained in the womb—the placenta. I told her that I massaged the womb, and the placenta popped out. She helped me by preparing water and I showered. She prepared some porridge and went away. (Age 20–29 years, two children)

Some support persons also helped women access healthcare, including calling TBAs, escorting women to TBAs and/or accompanying women to the hospital after they gave birth.

After giving birth, they [friend and husband] cut the umbilical cord, wrapped him [baby] in warm clothing, and then we went to the hospital. (Age 20–29 years, two children)

A few women reported that their support persons provided encouragement and emotional support, as well as useful instructions during labour and birth. Notably, no women described experiencing harassment by their friends or family members.

There is one [friend] who was rubbing my back…Another one [friend] was telling me to open my legs so that I do not obstruct the baby…They took care of me. (Age 30–39, four children)

As with women who gave birth with TBAs, many women who gave birth with support persons expressed that they felt worried and stressed about experiencing potential complications during labour and birth.

It was difficult because when you are at the hospital and something is not right, the doctors can help you. They can take you to the theatre and see how to help you. But you see, I was alone between death and my God. (Age 20–29 years, four children)

Notably, many women were actually alone at the time they gave birth and received assistance from support persons or husbands only after the baby was born. These women managed to give birth, catch the baby and, in some instances, even cut the umbilical cord before any help arrived.

By the time they had arrived my water had already broken. I just prayed to God…And I pushed the baby and she came out and I held her with my hands. Then my husband went to knock at peoples’ gates looking for my friend. By the time that lady came, I was already holding the baby. Then she had to look for a razor, and the cold was getting to the child and myself…I was very cold. I was shivering. (Age 30–39 years, two children)

Finally, several women noted that their homes were not suitable environments for childbirth.

The difference [between giving birth at a healthcare facility versus at home] was that I didn’t give birth on the bed. I gave birth on the floor. I couldn’t give birth on the mattress, because there was no hospital mattress, and so I gave birth on the floor. (Age 30–39 years, four children)

While some women reported having cotton wool or razor blades with which to cut the umbilical cord, a few mentioned that they did not have these necessary supplies. One woman explicitly noted that she did not have these supplies because she had intended to deliver at a healthcare facility.

I had planned to deliver at the hospital. If I had planned to deliver at home, I would have bought the necessities like the razor blade. (Age 20–29 years, three children)

#### Husbands’ involvement during labour and birth

While several women noted that their husbands were present during labour and/or birth, most of the women assisted by support persons reported that their husbands did not directly provide any support. Generally, husbands’ involvement was limited to calling other support persons or arranging transportation for women to go to the TBA or healthcare facility. Overall, some women wanted their husbands to be present during labour and birth, even if they did not provide any direct support.

Yes, I would have liked [for husband to be involved]. At least you feel that somebody is in your life and you are together. When you are like that, you are also happy. (Age 20–29 years, four children)

However, a few women noted that they would not want their husbands to be present as they did not believe it was socially acceptable.

When giving birth, I wouldn’t want him to be around (laughs). It is not a good scene, what will he think? (Age 30–39 years, four children)

#### Complications of birth and delayed access to postpartum and postnatal healthcare

A few women who gave birth with support persons reported experiencing complications during and after birth, including the mother or infant catching a cold and excessive bleeding and tearing. As with women who gave birth with TBAs, women who gave birth with support persons faced delays in accessing postpartum and postnatal healthcare. More than half of these women did not access care for a week or more after giving birth. Most commonly, women delayed postpartum or postnatal care due to pain or weakness, while others delayed seeking care due to fear of judgement from healthcare providers.

I decided to wait for two days [to seek postpartum care] because I had so much pain in the stomach. I was unable to go because of the pain. (Age 40+ years, four children)

## Discussion

This study examined the experiences of women who unexpectedly gave birth outside of healthcare facilities in Kenya during the COVID-19 pandemic. In this sample, about one-third of women received labour and birth care from TBAs, and the majority were supported by friends, family members and/or husbands. Women primarily described aspects of their experience relating to the PCMC domain of supportive care, both in terms of care that they received (eg, emotional support) and that they did not receive but would have liked (eg, appropriate supplies and environment for giving birth). While no women in this sample described severe complications with birth, women experienced significant stress and worry about potential complications due to giving birth outside of a healthcare facility. Additionally, many women faced delays in accessing postpartum and postnatal care due to pain or bleeding in the days after giving birth and/or fear of judgement from healthcare providers. This study highlights notable gaps in PCMC that could be improved for women in the future.

The context of the COVID-19 pandemic likely influenced the level of person-centred care women in this study received during labour and birth. This study follows a prior study of this sample, which demonstrated that the COVID-19 pandemic resulted in these women giving birth outside of healthcare facilities, contrary to their initial plan for birth.^23^ The previous study found that these women gave birth outside of healthcare facilities because of fear of contracting COVID-19, lack of perceived community safety and fear of encounters with law enforcement during curfew hours and changes in access to the healthcare system due to the pandemic. Importantly, the previous study documented that almost all women in this sample had intended to give birth in healthcare facilities, but due to COVID-19 related barriers, eventually gave birth outside of healthcare facilities. Therefore, it is likely that the lack of preparation and women’s expectations for birth influenced the level of person-centred care these women experienced. Other studies in Kenya have found that because of over-stretched health systems during the COVID-19 pandemic, home births increased, often with the assistance of TBAs, who were stressed with the level of demand and lacked adequate support under the circumstances.^2023^,^25^ This stress may influence TBAs’ ability to provide high-quality, person-centred care. Moreover, the current study also found that access to timely postpartum care was low, which was likely exacerbated by the COVID-19 pandemic. Other studies have similarly found that women in Kenya delayed or avoided necessary maternal health services during the pandemic.^1314 18^,^22^

Although this sample was unique in that these women did not initially intend to give birth outside of healthcare facilities, the findings regarding women’s experiences with TBAs corroborate and expand upon prior literature. Of the women who were assisted by TBAs, most selected the TBA based on a prior relationship with the TBA or personal recommendations from other women in their community. This aligns with the existing literature indicating that TBAs are generally known in the community for the services they provide and are trusted by women.^28 34 35 42^ Additionally, in this study, women described aspects of their experience with TBAs that reflect the PCMC domains of dignity and respect as well as supportive care, including encouragement, checking in with women during labour, completing clinical tasks such as cutting the umbilical cord and providing instrumental support such as preparing food or baths. Prior literature has emphasised that care from TBAs is particularly appealing for women as TBAs may provide emotional and instrumental support, which are less commonly provided in healthcare facilities.^3240^,^42^

However, many women in this study also described aspects of their experience with TBAs that were not person-centred, particularly regarding the domains of dignity and respect and supportive care. As in prior studies,^32 35 41 42^ women in this study emphasised that TBAs were unable to address medical complications, which made women worry about adverse outcomes for themselves and their infants. Some women in this study also stated that the environment in which they gave birth with TBAs was not hygienic nor private, which has been noted previously.^28 35^ Though rare in this study, a few women also reported that they faced verbal and physical harassment by TBAs. These gaps in PCMC reinforce the drawbacks of giving birth with TBAs and suggest some opportunities to improve the quality of labour and birth care provided by TBAs.

Among women who were supported by friends, family members and/or husbands, these support persons most commonly helped women by providing encouragement during labour and cutting the umbilical cord after birth, as well as helping women access healthcare for birth and the immediate postpartum. These actions are similar to those completed by support persons during births in healthcare facilities.^51^ Some women in the current study did not have proper supplies available at home, such as a sheet on which to give birth or a razor with which to cut the umbilical cord. In general, women’s husbands were not involved in labour and birth, which aligned with some women’s preferences, while others would have liked their husbands to be involved to some extent. Given the unexpected nature of labour for many of these women, women generally described being grateful for any support in the moment. Notably, in a prior survey of women who gave birth in healthcare facilities in Kenya, most wanted to have companions present during labour, but less than half wanted to have companions present during delivery.^52^ In the current study, several women were actually alone when they gave birth and were assisted only in the period immediately following birth. This aligns with findings from an analysis of women delivering in Kenya during the COVID-19 pandemic, which found that less than 10% of women had companions present during labour and delivery in healthcare facilities.^53^

### Limitations and strengths

This study has some important limitations. First, all but one of the women in this study intended to deliver in a healthcare facility, yet were unable to reach a healthcare facility while they were in labour.^23^ It is likely that women who intended to give birth outside of healthcare facilities, whether with TBAs or support persons, would have different experiences than those reported here. It may be that those who intend to give birth outside of healthcare facilities have better experiences than those reported here due to greater preparation for these births. Second, all participants in this study lived in Kiambu and Nairobi counties and, specifically, within the catchment area of the six participating health facilities. As such, these women’s experiences may differ from those who live in more rural regions and those who live further away from healthcare facilities. We would expect that women who live further from healthcare facilities may face even more challenges in accessing maternity healthcare, including delivering at healthcare facilities. Third, this study was not specifically designed to examine the domains of PCMC. The interview field guide asked women about their experiences during pregnancy, birth and postpartum, but did not probe specifically on each PCMC domain. This is likely why no themes were identified related to the communication and autonomy domain of PCMC. It is possible that aspects of labour and birth care relating to this domain were actually salient for women, but were not mentioned during interviews as women were not asked about this directly. Future research should thoroughly explore each PCMC domain to investigate the quality of labour and birth care women receive outside of healthcare facilities. Finally, this study focused primarily on the labour and birth experience, with less emphasis on the postpartum and postnatal periods. Given the importance of care in the days and weeks after giving birth, future studies should explore how giving birth outside of healthcare facilities and, particularly with TBAs, is related to access to postpartum and postnatal care.

Despite these limitations, this study has notable strengths. First, the data were collected early in the COVID-19 pandemic in Kenya. Therefore, these women can uniquely describe the experience of giving birth during a period of acute strain on the healthcare system within the context of the COVID-19 pandemic. This study provides a unique opportunity to examine experiences among women who were unable to reach a healthcare facility during labour and thus did not give birth in their preferred setting. Notably, these findings may have implications for other public health emergencies in the future. As emergencies including infectious disease pandemics are likely to occur again in the future, it is helpful to understand women’s experiences giving birth during these emergencies to better prepare the healthcare system to meet the needs of pregnant women in these scenarios. Finally, this is the first study to our knowledge to examine women’s experiences giving birth outside of healthcare facilities through the lens of PCMC. As such, this study provides an important initial examination of the extent to which women’s experiences were person-centred and identifies ways in which TBAs could provide more person-centred care.

### Implications for Policy and Future Research

Understanding women’s experiences giving birth outside of healthcare facilities during the COVID-19 pandemic sheds light on vulnerabilities within the healthcare system during emergencies. It is vitally important to invest in maternity healthcare services during emergencies to ensure both maternal and neonatal safety.^54^ Notably, several efforts existed in Kenya during the COVID-19 pandemic to facilitate access to healthcare facilities for pregnant women. In particular, the MomCare digital platform provided information about available maternity care services,^55^ while Wheels of Life provided free transportation to hospitals for pregnant women.^56^ Despite these initiatives, many women were unable to access health facilities while in labour, leaving them to unexpectedly give birth outside of healthcare facilities, such as those women in the current study.^23^ Given that emergencies like the COVID-19 pandemic are likely to occur again in the future, additional policies and programmes are necessary to ensure pregnant women can access person-centred healthcare for labour and birth. These efforts should not only address the identified barriers to facility-based births,^23^ but should also better prepare women and TBAs to manage labour and births outside of healthcare facilities in case of unexpected circumstances.

This study suggests that when women face disruptions in healthcare services which prevent them from giving birth at a facility, they may turn to TBAs for support. Indeed, even in non-emergency situations, deliveries with TBAs are common.^29^ With appropriate support, TBAs could be equipped to provide more PCMC for the women they serve both during and outside of emergency situations.^24^ This study found that women expressed concerns regarding some aspects of their experience with TBAs, such as lack of privacy and hygiene in the birthing environment and some instances of harassment. These gaps in person-centred care could be addressed through providing TBAs with additional training and necessary equipment. Women also reported significant worry and stress given that TBAs could not address medical complications that may arise. Providing opportunities for training and capacity-building of TBAs as well as streamlined processes for TBAs to refer and transfer high-risk patients to healthcare facilities may help mitigate these fears and ensure complications during labour and birth are addressed. Additionally, women in this study reported delays in seeking postpartum and postnatal services. As suggested in prior literature, TBAs could be more integrated within the broader healthcare system to encourage continuity of care between birth and the postpartum/postnatal period.^24^ Given the fear of judgement by healthcare providers that women indicated prevented them from accessing needed postpartum and postnatal care in the current study, facility-based healthcare providers could be more welcoming to women into postpartum and postnatal care without judgement for the circumstances of their births. Finally, more research is needed to better understand women’s experiences giving birth with TBAs and to develop culturally appropriate interventions to improve person-centred care from TBAs. In particular, future studies should consider adapting the PCMC scale for this setting and directly measuring each domain of PCMC. Although women are recommended to give birth at healthcare facilities in Kenya, women who turn to TBAs for any reason still deserve to receive PCMC that is respectful of their needs and preferences.

### Conclusion

This study examined the experiences of women in Kenya who gave birth outside of healthcare facilities during the COVID-19 pandemic. When women were unable to access health facilities for their birth, they turned to TBAs, friends, family members and/or husbands for support. While TBAs and support persons helped women navigate these unplanned circumstances, many women were unprepared both materially and psychologically to give birth in these settings. Women primarily described salient aspects of their labour and birth experiences with TBAs relating to the PCMC domain of supportive care. Although many women described some aspects of person-centred care they received, many women did not receive person-centred care during birth. Most women in this study experienced stress and worry about potential medical complications and faced delays in accessing postpartum and postnatal healthcare. Future research should build on these findings to further explore levels of PCMC women experience when giving birth with TBAs and to identify appropriate interventions to improve PCMC for women who give birth outside of healthcare facilities, particularly during emergencies such as pandemics.

## Supplementary material

10.1136/bmjopen-2025-101458 online supplemental file 1

## Data Availability

Data are available upon reasonable request.
