# Supplementary material for: Women’s experiences giving birth outside of health facilities in Kenya during the COVID-19 pandemic: a qualitative study
Source: BMJ Open. 2025 Sep 15;15(9):e101458. doi: 10.1136/bmjopen-2025-101458 (PMC12439143; doi:10.1136/bmjopen-2025-101458)
Supplement: online supplemental file 1 [file bmjopen-15-9-s001.docx]

**Supplementary Materials**

**Table S1. Consolidated Criteria for Reporting Qualitative Research (COREQ) Checklist)***

| **Item** | **Guiding Questions/Description** | **Page Number** |
| --- | --- | --- |
| **Domain 1: Research team and reflexivity** | | |
| 1. Interviewer/facilitator | Which author/s conducted the interview or focus groups? | NA (Interviewer is not an author) |
| 1. Credentials | What were the researcher’s credentials? E.g. PhD, MD | 5-6 |
| 1. Occupation | What was their occupation at the time of the study? | 5-6 |
| 1. Gender | Was the researcher male or female? | 5-6 |
| 1. Experience and training | What experience or training did the researcher have? | 5-6 |
| *Relationship with participants* | | |
| 1. Relationship established | Was a relationship established prior to study commencement? | 5-6 |
| 1. Participant knowledge of the interviewer | What did the participants know about the researcher? E.g. personal goals, reasons for doing the research | 5-6 |
| 1. Interviewer characteristics | What characteristics were reported about the interviewer/facilitator? E.g. bias, assumptions, reasons and interests in the research topic | 5-6 |
| **Domain 2: Study design** | |  |
| *Theoretical framework* | | |
| 1. Methodological orientation and theory | What methodological orientation was stated to underpin the study? E.g. grounded theory, discourse analysis, ethnography, phenomenology, content analysis | 5-6 |
| *Participant selection* | | |
| 1. Sampling | How were participants selected? E.g. purposive, convenience, consecutive, snowball | 5-6 |
| 1. Method of approach | How were participants approached? E.g. face-to-face, telephone, mail, email | 5-6 |
| 1. Sample size | How many participants were in the study? | 5-6 |
| 1. Non-participation | How many people refused to participate or dropped out? Reasons? | 5-6 |
| *Setting* | |  |
| 1. Setting of data collection | Where was the data collected? E.g. home, clinic, workplace | 5-6 |
| 1. Presence of non-participants | Was anyone else present besides the participants and researchers? | 5-6 |
| 1. Description of sample | What are the important characteristics of the sample? E.g. demographic data, date | 5-6 |
| *Data collection* | | |
| 1. Interview guide | Were questions, prompts, guides provided by the authors? Was it pilot tested? | 5-6 |
| 1. Repeat interviews | Were repeat interviews carried out? If yes, how many? | NA (no repeat interviews) |
| 1. Audio/visual recording | Did the research use audio or visual recording to collect the data? | 5-6 |
| 1. Field notes | Were field notes made during and/or after the interview or focus group? | 5-6 |
| 1. Duration | What was the duration of the interview or focus group? | 5-6 |
| 1. Data saturation | Was data saturation discussed? | 5-6 |
| 1. Transcripts returned | Were transcripts returned to participants for comment and/or correction? | NA (transcripts not returned to participants) |
| **Domain 3: Analysis and findings** | | |
| *Data analysis* | | |
| 1. Number of data coders | How many data coders coded the data? | 5-6 |
| 1. Description of the coding tree | Did authors provide a description of the coding tree? | 5-6 |
| 1. Derivation of themes | Were themes identified in advance or derived from the data? | 5-6 |
| 1. Software | What software, if applicable, was used to manage the data? | 5-6 |
| 1. Participant checking | Did participants provide feedback on the findings? | NA (participants were not asked for feedback on findings) |
| *Reporting* | |  |
| 1. Quotations presented | Were participant quotations presented to illustrate the themes/findings? Was each quotation identified? E.g. participant number | 6-11 |
| 1. Data and findings consistent | Was there a consistency between the data presented and the findings? | 6-11 |
| 1. Clarity of major themes | Were major themes clearly presented in the findings? | 6-11 |
| 1. Clarify of minor themes | Is there a description of diverse cases or discussion of minor themes? | 6-11 |

*Questions from Tong et al., 2007.

**Table S2. Reflexivity on partnership between researchers from high-income and low-income/middle-income countries.***

| 1. **How does this study address local research and policy priorities?** | The study assesses challenges in maternity care that were experienced in Kenya during the COVID-19 pandemic and provides recommendations to address these challenges in future emergencies. The study team met with members from the Ministry of Health and included local researchers and partners to identify policy priorities for the study. |
| --- | --- |
| 1. **How were local researchers involved in study design?** | Local researchers were involved throughout the study beginning at the grant-application stage and contributed heavily to the study design. Two of these local researchers are co-authors on this manuscript. Local researchers advised on what was feasible in terms of data collection given the lockdown, made connections with local leaders to recruit participants, and determined the best approaches for recruitment. |
| 1. **How has funding been used to support the local research team(s)?** | Funding was used to cover the salaries of all local researchers who worked on the study, including those included as co-authors on this manuscript. Funding was also used to cover other work-related costs for communication, transportation, etc. |
| 1. **How are research staff who conducted data collection acknowledged?** | Two of the local researchers who conducted data collection are authors on this manuscript. Other researchers who contributed to data collection and analysis but did not work on this manuscript are listed in the acknowledgements. |
| 1. **How have members of the research partnership been provided with access to study data?** | Local researchers collected the primary data and de-identified the data consistent with the Kenya Data Protection Act. Local researchers then shared the de-identified data with the full team through an encrypted Box folder. Local researchers participated in the cleaning and analysis of study data. |
| 1. **How were data used to develop analytical skills within the partnership?** | The research team, including those from Kenya and the U.S., collaboratively analyzed data for this study. During the analysis process, Kenyan researchers were trained in qualitative coding techniques and use of Dedoose software. |
| 1. **How have research partners collaborated in interpreting study data?** | The researchers who coded the interviews described in this manuscript included the local researcher who conducted interviews and two other local researchers involved in data collection, including the project supervisor in Kenya. These researchers were integral throughout the coding and analysis process to ensure accurate interpretation of data. Multiple meetings were held to discuss and interpret study findings with researchers in both Kenya and the U.S. throughout the coding and analysis process. |
| 1. **How were research partners supported to develop writing skills?** | Research partners contributed to the writing and revision of this manuscript, and gained both expertise and mentorship on writing skills. |
| 1. **How will research products be shared to address local needs?** | Findings from both these interviews described in this manuscript and the quantitative parent survey were shared with the Kenyan Ministry of Health in an oral presentation and a policy brief. Additionally, several manuscripts using the data from this study have been published in open-access journals to increase access to study findings in Kenya. |
| 1. **How is the leadership, contribution, and ownership of this work by LMIC researchers recognized within the authorship?** | The authors of this manuscript include researchers from both the U.S. and Kenya. Two of the authors of this manuscript are research partners located in Kenya, while three of the authors are located in the U.S. |
| 1. **How have early career researchers across the partnership been included within the authorship team?** | The authorship team includes three early career researchers, two of whom are currently enrolled in a doctoral program and one of whom is currently applying for a doctoral program. |
| 1. **How has gender balance been addressed within the authorship?** | The authors include four cisgender women and one cisgender man. |
| 1. **How has the project contributed to the training of LMIC researchers?** | Kenyan researchers gained training throughout the entire study process, including participant recruitment, data collection, data cleaning, data analysis, and manuscript drafting and revision. |
| 1. **How has the project contributed to improvements in local infrastructure?** | Kenyan researchers applied their skills through contribution to this research. This is consistent with the goal to increase capacity among local researchers to steer research and research agendas. This experience prepares researchers to eventually conduct a study as a lead researcher in the future. While this study was exploratory in nature, it points to important insights into home birth experiences during a global pandemic. Application of these findings and additional research may lead to more inclusive, person-centered care for women who give birth outside of healthcare facilities. |
| 1. **What safeguarding procedures were used to protect local study participants and researchers?** | All study procedures were reviewed and approved by the Institutional Review Board at the University of California, Los Angeles as well as the Kenya Medical Research Institute. |

*Questions from Morton et al., 202

**Field Guide for In-Depth Interviews**

**Demographics**

1. How old are you?
2. Was this your first birth?
   1. If not, how many children do you have?
3. Are you currently married/partnered?
4. Who lives in your house with you? [Probe: Children vs. adults]

**COVID-19**

1. Have you known anyone who ever had, or thought they had, COVID-19?
2. Have you ever had, or thought you might have had, COVID-19?
   1. How did you react?
   2. Did you seek any information or help? If so, from whom/where?
   3. Did you get tested?
3. To what extent has COVID-19 impacted you?
4. What resources have you received for COVID-19? [Probe: Financial, social, health]
5. How did you learn about the government procedures related to COVID-19 (stay at home orders, policies wearing face masks, curfews, transport restrictions, etc.)?
6. Where or from whom do you get your COVID-19 related news, information, and resources?
7. How has language accessibility or inaccessibility of COVID-19 related awareness materials affected you/friends/family?
8. How satisfied have you been with the national government in responding to COVID-19? How about the county government?

**Pregnancy and Delivery**

Next, I would like to hear about your experiences with your most recent birth.

1. What was the most challenging thing about being pregnant during this time of COVID-19?
2. Did you go for antenatal care visits?

*If attended ANC:*

- 1. Where did you go? Why did you choose to go to this place?
  2. When did you go for ANC during your pregnancy? Why did you decide to go at that time?
  3. What did you think about your experience during your antenatal care visits?
     1. How did you feel about the services that you received? Why?
     2. How did you feel about the staff that attended to you? Why?
  4. During your antenatal care visits, what information did your health provider talk to you about?
  5. Did your provider talk to you about where you plan to deliver your baby? What did you discuss during this conversation?

*If did not attend ANC:*

- 1. Why did you choose not to go for any antenatal care?

Next, we are interested in your experiences with delivering at home.

1. How did you choose to deliver at home?
   1. Who helped you to make decisions about where to deliver?
      1. What role did they have in making these decisions?
   2. Did the orders to stay at home/curfews due to COVID-19 influence your decision to deliver at home? How so? [Probe: Safety, transportation, other]
2. Did you always plan to deliver at home?

*If not:*

- 1. Where did you originally plan to deliver?
     1. Why did you plan to deliver there?
  2. What were the reasons you delivered at home instead?

*If this is* ***not*** *her first birth/child:*

- 1. Where did you deliver your other children?
     1. Why did you go to that place for your other deliveries?

*If different location than most recent delivery:*

- 1. Why did you decide to instead deliver at home for this delivery?
  2. How was your experience this time different than your previous experience(s)?

1. Can you describe your overall experience delivering at home?
   1. How do you feel about this experience?
2. Who was present with you during your delivery?
3. Why did you have these people present with you?
4. What did they do during your delivery?

*If trained birth attendant (TBA) present:*

1. Why did you choose to have this person present with you?
2. How did you select this person?
3. What is your relationship to the TBA? How long have you known the TBA?
4. What did the TBA do during your delivery?
   - 1. What was their role? How did they help you?
5. How do you feel about their role during your delivery?
   - 1. How satisfied were you with their assistance? Why?

*[ALL]:*

1. Who did you turn to for advice and information about your pregnancy?
   1. Why did you ask these people?
   2. How did they help you?
   3. Did you speak to anyone about where you planned to delivery your baby?
      1. What did you discuss with this person?
2. *[If married, partnered]* What role did your husband or partner play in your decisions about pregnancy and delivery?
   1. What did you and your husband or partner discuss about where to go for delivery?
   2. How did you feel about your husband’s or partner’s involvement during these decisions?
   3. What role did your husband or partner have during your delivery? What about after delivery?
   4. How did you feel about his role? Or support?
3. Have you ever experienced an unexpected health problem or complication during any of your pregnancies or deliveries?

*If yes:*

- 1. Please tell me about what happened.
     1. What did you do?
     2. Where did you go?
     3. Who helped you to go there?
     4. Was the health problem/complication cured/resolved?
  2. Were you told to go to another place (i.e., referred to another clinic or hospital)?
     1. Who told you to go?
     2. Where did they tell you to go? Why did they tell you to go there?
     3. Did you go?
     4. Who made the decision for you to go to that place?

1. What was the most challenging thing about delivering a child during COVID-19?

**Newborn Care**

1. How is your child doing [*referring to most recent birth*]? How old are they now?
2. Have you been able to attend all your newborn visits? How many times have you visited the doctor?
   1. If you have not attended/skipped any newborn visits, why did you skip a visit?
   2. How do you access other sources of care? [Probe: Trained birth attendant, mobile clinics, etc.]
3. How has COVID-19 impacted health care for you or your child after you delivered?
4. What is the most challenging thing about caring for a newborn during COVID-19?
5. Do you feel safe going out in your community?
   1. Do you wear face masks? How often do you wear face masks (all of the time, just some of the time, or never)? Why?
   2. Do you use hand sanitizer when outside of your home? Why or why not?
   3. Do you have adequate transportation to see the doctor?

Finally, what recommendations do you have to improve your experiences with your pregnancy or your delivery during COVID-19/the coronavirus pandemic?

Is there anything else you’d like to share about your experience before we end the interview?
