# Supplementary material for: Women’s experiences giving birth outside of health facilities in Kenya during the COVID-19 pandemic: a qualitative study
Source: BMJ Open. 2025 Sep 15;15(9):e101458. doi: 10.1136/bmjopen-2025-101458 (PMC12439143; doi:10.1136/bmjopen-2025-101458)
Supplement: Supplementary file 2 [file bmjopen-2025-101458.reviewer_comments.pdf]

PEER REVIEW HISTORY

BMJ Open publishes all reviews undertaken for accepted manuscripts. Reviewers are asked to complete a checklist review form and are provided with free text boxes to elaborate on their assessment. These free text comments are reproduced below.

ARTICLE DETAILS

Title (Provisional)

Women's Experiences Giving Birth Outside of Health Facilities In Kenya During the COVID-19 Pandemic: A Qualitative Study

Authors

Woofter, Rebecca; Varghese, Krupa; Mboya, John; Golub, Ginger; Sudhinaraset, May

---

VERSION 1 - REVIEW

**Reviewer**	**1**
**Name**	**Spigel, Lauren**
**Affiliation**	**Ariadne Labs at Brigham and Women's Hospital and the Harvard T.H. Chan School of Public Health**
**Date**	**09-May-2025**
**COI**	**None**

The authors addressed an understudied topic: experience of women giving birth outside of health facilities in Kenya. This is an important area of research and the authors did a commendable job at giving voice to this often invisible issue.

I have a few comments that I hope authors will find helpful. My comments are organized by section.

Contributions section:

KS drafted the manuscript with RW but is not listed as an author. Maybe a typo?

Abstract:

The PCMC acronym needs be be defined in the abstract. Please also include more about the study design.

Methods:

Thank you for including COREQ.

Please say more about the study design. It sounds like it may be a descriptive study. If you agree, I find this reference helpful: Sandelowski M. Whatever happened to qualitative description? Res Nurs Health. 2000 Aug;23(4):334–40.

Eligibility criteria: Were any of the survey participants who gave birth outside of a facility ineligible for the study due to giving birth prior to March 16, 2020?

Please address saturation. Was saturation an intended goal of this study? If so, what kind of saturation? Was saturation reached? How was saturation assessed? I find these sources helpful:

Saunders B, Sim J, Kingstone T, Baker S, Waterfield J, Bartlam B, et al. Saturation in qualitative research: exploring its conceptualization and operationalization. Qual Quant. 2018;52(4):1893–907.

Hennink MM, Kaiser BN, Marconi VC. Code saturation versus meaning saturation: how many interviews are enough? Qual Health Res. 2017 Mar;27(4):591–608.

It would be helpful to include the interview guide and codebook as an appendix.

Authors used inductive coding. For their thematic analysis, they drew on the PCMC framework. Please describe the process used to progress from inductive codes to themes around this specific framework.

Please also elaborate on how the PCMC framework was used in the collection, analysis and/or interpretation of the data.

Results are stratified by those who were assisted by TBAs and by those assisted by support persons. This seems like a reasonable approach, but authors should describe the stratified analysis in the methods section.

Results:

Please provide a high-level overview of the main themes that came out of the analysis before sharing results of each theme.

It would be helpful to see a table of PCMC domains with a description of how each domain was defined for this research, including how the themes mapped to the PCMC framework. It may also be helpful to add a summary of results for each domain in the table as well.

Discussion

I'd like more context around COVID-19. Are authors assuming that most of these births would have happened in a hospital had it not been for COVID-19? (For example, transportation issues, precipitous labor, other barriers to getting to care can happen outside of a pandemic context). How did COVID-19 itself affect the experience of giving birth outside of the facility? How do these results compare to results of similar studies both during COVID-19 and not during COVID-19? In other words, is there anything unique about these results because of COVID-19? There didn't seem to be much mention of COVID-19 in the results,

which is interesting, as this study is framed as taking place after COVID-19 policies were implemented. Were study participants asked about how COVID-19 affected their birth experience? If so, it might be worth adding some of that information into the results section, and then elaborating on it in the discussion. If not, the paper would still benefit from a discussion about COVID-19, especially because authors frame these results as useful to inform maternity care during other emergencies.

**Reviewer**	**2**
**Name**	**Ombere , Stephen Okumu**
**Affiliation**	**Maseno University, Sociology and Anthropology**
**Date**	**19-May-2025**
**COI**	**None**

Dear Authors,

Thank you for taking the time to put this piece of writing together. The objective of this study is still relevant and contributes to the current debate on safe motherhood in Kenya during the pandemic.

I have a few issues that you need to attend to. They can help improve this manuscript.

-Write PCMC in full on first usage in the abstract.

--In the abstract, please mention the design.

Introduction:

-Due to the current debate on birth and birthing processes and safe-motherhood, could the authors in the entire manuscript use traditional midwives instead of traditional birth attendants or use the two interchangeably but with a disclaimer from the word go that the two mean the same thing.

-Could the authors introduce who TBAs or traditional midwives are in the context of this study? This can come in the last section of the second paragraph.

-Delete the word 'unexpectedly' in line 53 of the introduction.

-I have a problem with this statement...'However, no studies to date have examined......' could the authors re-word/rephrase this to expose a research gap for this study? I am aware studies have been done to this effect, but there are still issues that are worth researching on some of which this article articulates very well

-Justify the choice of the two study areas and not Wajir or Mombasa in Kenya.

-Findings are fairly presented. However, I feel the researchers did not probe some of the participants i.e. after TBAs assistance, what next? Did care end at delivery? Were they later referred to some biomedical facilities? Otherwise, as the findings stand, this article is skewed

towards advocating for home births, a thing that is not permitted in Kenya. Think about the implications of these findings.

-Discussion and study limitations are to the point.

I look forward to reading this article soon.

VERSION 1 - AUTHOR RESPONSE

Reviewer 1	
Contributions section:<br>KS drafted the manuscript with RW but is not listed as an author. Maybe a typo?	We have fixed this typo.
Abstract:<br>The PCMC acronym needs be be defined in the abstract. Please also include more about the study design.	We have written out the PCMC acronym. We have also described the study design further.
Please say more about the study design. It sounds like it may be a descriptive study. If you agree, I find this reference helpful: Sandelowski M. Whatever happened to qualitative description? Res Nurs Health. 2000 Aug;23(4):334–40.	This is a descriptive qualitative study. We have specified this and included the citation in the Methods section.
Eligibility criteria: Were any of the survey participants who gave birth outside of a facility ineligible for the study due to giving birth prior to March 16, 2020?	The survey was only open to those who gave birth on or after March 16, 2020, so we do not have delivery location information for those who gave birth prior to that date. We clarified this in the Methods section.
Please address saturation. Was saturation an intended goal of this study? If so, what kind of saturation? Was saturation reached? How was saturation assessed? I find these sources helpful:<br>Saunders B, Sim J, Kingstone T, Baker S, Waterfield J, Bartlam B, et al. Saturation in qualitative research: exploring its conceptualization and operationalization. Qual Quant. 2018;52(4):1893–907.<br>Hennink MM, Kaiser BN, Marconi VC. Code saturation versus meaning saturation: how many interviews are enough? Qual Health Res. 2017 Mar;27(4):591–608.	This study did not specifically aim to achieve saturation in the interviews. The number of interviews conducted was based on the highest number of interviews possible with the available resources. During coding, most codes were developed in the first phase (using 6 transcripts), with some added in the second phase (using 15 transcripts) and very few added in the third phase (using 7 transcripts). During thematic analysis, we identified commonalities across the transcripts particularly regarding experiences during labor and birth. Taken together, this suggests that saturation in terms of codes and themes was met. We have added these details and citations in the Methods section.
It would be helpful to include the interview guide and codebook as an appendix.	We have included the interview guide as an appendix. We have also added more

	description of the codebook in the methods section.
Authors used inductive coding. For their thematic analysis, they drew on the PCMC framework. Please describe the process used to progress from inductive codes to themes around this specific framework.<br><br>Please also elaborate on how the PCMC framework was used in the collection, analysis and/or interpretation of the data.	We have included more description about how the inductive codes were applied to the PCMC framework in the analysis section within the Methods.<br><br>We also noted that while the interviews were not specifically designed to capture the domains of PCMC, we determined after inductive coding that many of the codes related to the PCMC domains, and thus used this framework for analyses.
Results are stratified by those who were assisted by TBAs and by those assisted by support persons. This seems like a reasonable approach, but authors should describe the stratified analysis in the methods section.	We have added this to the methods section.
Results: Please provide a high-level overview of the main themes that came out of the analysis before sharing results of each theme.	We have added an overview of the main themes at the beginning of the Results section.
It would be helpful to see a table of PCMC domains with a description of how each domain was defined for this research, including how the themes mapped to the PCMC framework. It may also be helpful to add a summary of results for each domain in the table as well.	We have added a table with descriptions of the PCMC domains and examples for the domains from the results. We also noted that the field guide was not specifically developed to capture each domain of PCMC, which likely explains why we did not identify any themes relating to the communication and autonomy domain. We have also noted this in the limitations section.
Discussion: I'd like more context around COVID-19. Are authors assuming that most of these births would have happened in a hospital had it not been for COVID-19? (For example, transportation issues, precipitous labor, other barriers to getting to care can happen outside of a pandemic context).<br><br>How did COVID-19 itself affect the experience of giving birth outside of the facility? How do these results compare to results of similar studies both during COVID-19 and not during COVID-19? In other words, is there anything unique about these results because of COVID-19? There didn't seem to be much mention of COVID-	We have added a paragraph in the Discussion on how the COVID-19 pandemic likely influenced women's experiences of care. We describe our previous study on this sample which found that COVID-19 directly contributed to the home births in this sample and also other relevant literature in Kenya on how this may influence person-centered care.<br><br>Notably, although women indicated that COVID-19 was the reason they did not give birth at healthcare facilities, they did not mention the pandemic influencing their experience of labor and birth otherwise. This is why COVID-19 is not directly discussed in the Results section. We have

19 in the results, which is interesting, as this study is framed as taking place after COVID-19 policies were implemented. Were study participants asked about how COVID-19 affected their birth experience? If so, it might be worth adding some of that information into the results section, and then elaborating on it in the discussion. If not, the paper would still benefit from a discussion about COVID-19, especially because authors frame these results as useful to inform maternity care during other emergencies.	added more in the Discussion section about the role of COVID-19. It is likely that women who intend to give birth at home and/or with TBAs (either during COVID-19 or outside of that context) are more prepared for the births, and have different experiences than those presented here. We have noted this in the Limitations section.
Reviewer 2	
-Write PCMC in full on first usage in the abstract.	We have written out PCMC in the abstract.
--In the abstract, please mention the design.	We have described the study design further in the abstract.
Introduction:<br>-Due to the current debate on birth and birthing processes and safe-motherhood, could the authors in the entire manuscript use traditional midwives instead of traditional birth attendants or use the two interchangeably but with a disclaimer from the word go that the two mean the same thing.	We have described both traditional midwives and traditional birth attendants in the introduction. In the introduction, we use these terms interchangeably except when citing papers that specifically pertain to one or the other.<br><br>In this paper, we use the term TBA when describing the results because the participants referred to TBAs specifically, and the role of these individuals align with the definition of TBA.
-Could the authors introduce who TBAs or traditional midwives are in the context of this study? This can come in the last section of the second paragraph.	We have added more description of the role of traditional midwives and TBAs.
-Delete the word 'unexpectedly' in line 53 of the introduction.	We have removed this word.
-I have a problem with this statement...'However, no studies to date have examined......' could the authors re-word/rephrase this to expose a research gap for this study? I am aware studies have been done to this effect, but there are still issues that are worth researching on some of which this article articulates very well.	We have removed this sentence and clarified how our study adds to existing literature.
-Justify the choice of the two study areas and not Wajir or Mombasa in Kenya.	We conducted this study in Nairobi and Kisumu counties as these areas represent two large urban areas that are geographically close to each other, which helped with study logistics, particularly during the COVID-19 pandemic.

	Additionally, our study team has longstanding relationships with partners in these areas and have conducted quality improvement activities in healthcare facilities within these areas. We added a recommendation to the discussion to expand this work in other areas of Kenya, including Wajir and Mombasa.
-Findings are fairly presented. However, I feel the researchers did not probe some of the participants i.e. after TBAs assistance, what next? Did care end at delivery? Were they later referred to some biomedical facilities?<br><br>Otherwise, as the findings stand, this article is skewed towards advocating for home births, a thing that is not permitted in Kenya. Think about the implications of these findings.	This study was specifically focused on the experiences during labor and birth rather than postpartum. However, the field guide did include questions about the role of TBAs after the baby was born. Participants generally reported that TBAs did not provide care following birth. The only tasks participated stated that TBAs provided after the baby was born were cleaning the baby, preparing baths/food, and ensuring the placenta was delivered. Notably, these participants also did not see TBAs for antenatal care, so it is possible that the TBAs may offer postpartum care but the participants did not seek postpartum care with TBAs. We have noted in the limitations that future studies should focus more specifically on the postpartum period.<br><br>We have added more description in the discussion about the implications of our findings regarding births with TBAs. Studies suggest that both during COVID-19 and outside of the pandemic, some women give birth with TBAs for a variety of reasons. These findings suggest ways to improve the person-centered care TBAs provide women in either circumstance. We note in the Discussion that we build upon prior work highlighting the need to improve services from TBAs and integration of TBAs with healthcare facilities (e.g., Ombere 2021 in Frontiers in Sociology).

VERSION 2 - REVIEW

**Reviewer**	**1**
**Name**	**Spigel, Lauren**

Affiliation **Ariadne Labs at Brigham and Women's Hospital and the Harvard T.H. Chan School of Public Health**

Date **31-Jul-2025**

COI

This study adds an important perspective to the literature by examining person-centered care among Kenyan women who intended to give birth in a health care facility but were unable to. The authors have made considerable improvements to their manuscript.

Reviewer **2**

Name **Ombere , Stephen Okumu**

Affiliation **Maseno University, Sociology and Anthropology**

Date **05-Aug-2025**

COI

Dear Authors

Thank you for addressing reviewers' comments. The article has greatly improved. However, there are minor corrections you need to make.

-For instance, in the abstract and in the main document, 'This study used data from cross-sectional descriptive' should read descriptive cross-sectional survey. then you state the methods used, such as in-depth interviews.

On the participants section in the abstract, delete the word 'during'.

-It is data analysis, not 'analyses in the methods.

-In methodology, please revise 'Data collection of the parent survey occurred between September and November 2020' for clarity.

-Table 1, is well presented and makes the manuscript more informative. Is there a verbatim for communication and autonomy? This can be inserted.

Thank you, and I wish you all the best. Looking forward to reading this manuscript if published online.

Good luck.

VERSION 2 - AUTHOR RESPONSE

**Reviewer Comment**	**Response**
Reviewer 2	

Thank you for addressing reviewers' comments. The article has greatly improved. However, there are minor corrections you need to make.<br>-For instance, in the abstract and in the main document, 'This study used data from cross-sectional descriptive' should read descriptive cross-sectional survey. then you state the methods used, such as in-depth interviews.	We have revised this sentence to be more clear. Since we did not use any data from the survey itself, we did not mention that in the abstract.
- On the participants section in the abstract, delete the word 'during'.	We have made this change.
-It is data analysis, not 'analyses in the methods.	We have made this change.
-In methodology, please revise 'Data collection of the parent survey occurred between September and November 2020' for clarity.	We have revised this sentence to be more clear.
-Table 1, is well presented and makes the manuscript more informative. Is there a verbatim for communication and autonomy? This can be inserted.	We did not identify any quotes that aligned with the communication and autonomy theme. We describe in the discussion this is likely due to the lack of specific questions in the field guide asking about this domain, and recommend future studies include this.
